# The Impact of Personalized Feedback Interventions by a Gambling Operator on Subsequent Gambling Expenditure in a Sample of Dutch Online Gamblers

**DOI:** 10.1007/s10899-022-10162-2

**Published:** 2022-11-09

**Authors:** Michael Auer, Mark D. Griffiths

**Affiliations:** 1 neccton Ltd, Muhlgasse 23, 9900 Lienz, Austria; 2 International Gaming Research Unit, Psychology Department, Nottingham Trent University, 50 Shakespeare Street, NG1 4FQ Nottingham, UK

**Keywords:** gambling, online gambling, problem gambling, The Netherlands, gambling operator interactions, gambling expenditure

## Abstract

Player protection has become an important area for the gambling industry over the past decade. A number of gambling regulators now require gambling operators to interact with customers if they suspect they are gambling in a problematic way. The present study provided insight on the impact of personalized feedback interventions (PFIs) on subsequent gambling behavior among a Dutch sample of real-world gamblers. *Nederlandse Loterij* (the national Dutch Lottery operator) provided access to a secondary dataset comprising tracking data from online casino and sports betting gamblers (N = 2,576) who were contacted either by e-mail or telephone between November 2021 and March 2022 if they showed signs of problematic gambling as identified using behavioral tracking software. Compared to matched controls (n = 369,961 gamblers), Dutch gamblers who received a PFI (via e-mail [n = 1876] or a telephone call [n = 700]) from the gambling operator had a significant reduction in amount of money deposited, amount of money wagered, number of monetary deposits, and time spent gambling in the 30 days after being contacted. Gambling frequency as measured by the number of gambling days did not change significantly after a PFI. Telephone calls did not lead to a significant larger reduction with respect to the aforementioned behavioral metrics. High-intensity players reduced their gambling behavior as frequently as low-intensity players, which means that the intervention’s success was independent of gambling intensity. The impact on subsequent gambling was the same across age groups and gender. The results of the present study are of use to many different stakeholder groups including researchers in the gambling studies field and the gambling industry as well as regulators and policymakers who can recommend or enforce that gambling operators utilize responsible gambling tools such as using PFIs to those who may be displaying problematic gambling behaviors as a way of minimizing harm and protecting gamblers.

**Supplementary Information:**

The online version contains supplementary material available at 10.1007/s10899-022-10162-2.

## Introduction

### Online Gambling and Problem Gambling

Gambling is an activity in which individuals stake money (or something of financial value) on an event in which the outcome is unknown in an attempt to gain more money (or something of greater financial value) (Griffiths, 1995). Individuals can gamble in offline brick-and-mortar establishments such as casinos, gambling halls, amusement arcades and betting shops or they can gamble online. Online gambling products are usually similar to land-based products and the only difference is the mode of access (Gainsbury, 2015). A number of previous studies have underlined the elevated risk of online gambling (e.g., Griffiths et al. 2006; Hubert & Griffiths 2018; McBride & Derevensky, 2009; McCormack et al., 2014).

Griffiths (2003) posited a number of situational factors in relation to online gambling that could lead to vulnerable individuals having increased gambling problems. Among the factors mentioned were high accessibility, anonymity, convenience, escape, immersion/dissociation, disinhibition, and interactivity. Moreover, online gamblers are usually able to select from a greater variety of games and play multiple games in parallel on the internet which has shown to be a risk factor for problematic gambling (Braverman et al., 2013; McCormack et al., 2014). In a review of the available literature Gainsbury (2015) concluded that online gambling did not cause gambling problems in, and of, itself. However, the review showed that online gambling was more common among highly involved gamblers, and for some online gamblers, this medium appeared to significantly contribute to gambling problems.

Chóliz et al. (2021) analyzed the prevalence of gambling disorder in Spain, as well as differences between online gambling (which was legalized in 2012) and traditional gambling, according to gender and age group. Chóliz et al. (2021) had access to the authorized databases of surveys carried out by the General Directorate of Gambling Regulation (Dirección General de Ordenación del Juego, 2016). They found that there were differences between age groups with respect to gambling involvement but not with respect to the prevalence of pathological gambling. A total of 12.5% of people younger than 26 years had gambled online compared to 56% who had participated in any type of gambling. Females had a significantly lower prevalence of pathological gambling than males among all age groups, indicating that gender is a particularly relevant variable in the prevalence of gambling disorder. The prevalence of pathological gambling among gamblers who had gambled online was 7.26%, whereas it was 0.69% among those who had not. Pathological gambling occurred among gamblers who also gambled online at a frequency 10 times higher than in gamblers who had not gambled online. Similar findings were also reported by Griffiths et al. (2009) using data from a nationally representative British sample (5% problem gambling prevalence rate among those who gambled online compared to 0.5% who did not). However, it must be noted that almost all online gamblers also gamble offline and that these gamblers should be described as ‘multi-modal’ gamblers rather than online gamblers. In fact, one study using a nationally representative sample of British gamblers (i.e., Wardle et al., 2011) reported no cases of problem gambling among those participants who only gambled online and that the highest prevalence of problem gambling was among multi-modal gamblers (4.3%) followed by those who gambled offline only (0.9%).

Researchers have also found that adolescents are vulnerable to developing online gambling problems (e.g., Gainsbury 2015; Hubert & Griffiths, 2018). Part of the explanation involves the developmental characteristics of adolescence, which is a period of particular vulnerability to engage in multiple forms of risky behavior (Jessor, 1991) and develop addiction problems due to immature self-regulation capacity, impulsivity, external locus of control, and susceptibility to contextual factors (Hollén et al., 2020). Recent studies have also demonstrated a significant increase in online gambling behavior among females, as well as changing trends in online gambling problem development (Hollén et al., 2020; McCormack et al., 2014; Volberg et al., 2018).

### Responsible Gambling

Only 5–10% of individuals who develop gambling problems seek treatment (Slutske, 2006). Preventing problem gambling is as important as it is to provide treatment. Besides restrictions in gambling availability, promotion of responsible gambling is seen as a strategy for preventing gambling problems (Williams et al., 2012). In previous years, a number of studies have been published with regard to the prevention of problem gambling among online gamblers (e.g., Edgerton et al., 2016; Haefeli et al., 2011; Shi et al., 2021).

Personalized feedback is one responsible gaming tool which has been subject to several studies using real gamblers on actual gambling websites (e.g., Auer & Griffiths 2014, 2015a, 2016, 2018, 2020). Researchers have hypothesized that gamblers are not able to keep track of their gambling, especially for games with a high event frequency. Auer and Griffiths (2017) compared gambler’s actual behavioral tracking data with their self-report data over a one-month period. A total of 1335 Norwegian gamblers answered survey questions relating to their gambling expenditure that was then compared with their actual gambling expenditure. They found that the estimated loss self-reported by gamblers was correlated with the actual objective loss but that gamblers with higher losses tended to have much more difficulty and were much less reliable in estimating their gambling expenditure. They concluded that feedback concerning actual spending is an important responsible gaming strategy.

Wohl et al. (2017) asked 607 Canadian gamblers who had enrolled in a casino-loyalty program how much they had won or lost over a three-month period while using their loyalty card. Results indicated that gamblers who under-estimated their losses significantly reduced the amount they wagered as well as the amount they lost during a follow-up period. In a sample of 1,015 online gamblers, Auer and Griffiths (2015a) found that gamblers who received feedback about money and time spent significantly reduced subsequent gambling expenses. A follow-up study with a population of Swedish gamblers supported the findings (Auer & Griffiths, 2020). Two studies with real-world online gamblers have also shown that normative feedback about other gamblers’ expenditure also appears to reduce subsequent gambling behavior (Auer & Griffiths, 2015b, 2016).

In recent years the effectiveness of voluntary vs. mandatory responsible gaming tools has increasingly been discussed. Ivanova et al. (2019) evaluated the impact of deposit limit prompts on the frequency of voluntary deposit limit setting among a sample of Swedish online gamblers. They reported that prompting online gamblers to set a voluntary deposit limit of optional size did not affect subsequent net loss compared to unprompted customers. Consequently, they concluded that voluntary limits are not an adequate responsible gaming tool because only a small percentage of players use them. Delfabbro and King (2021) conducted a review of mandatory vs. voluntary limit-setting research. They concluded general support for the potential benefits of mandatory systems. They also highlighted some potential selective uses for voluntary systems while also noting potential risks associated with implementing mandatory global limits. In a commentary on Delfabbro and King’s (2021) review, Auer and Griffiths (2021) noted that only ten of the 25 listed studies by Delfabbro and King were published in peer-reviewed journals and given the high reliance on studies in the grey literature, there were other studies that could have been included. Auer and Griffiths (2021) also listed other studies meeting Delfabbro and King’s inclusion criterion that could have provided further useful data.

Peter et al., (2019) reviewed 11 studies with respect to the effect of personalized feedback. They concluded that interventions appeared to be most effective when (i) used among populations of greater gambling severity, (ii) individuals were provided with gambling-related educational information, and (iii) used in conjunction with motivational interviewing. Factors associated with reduced efficacy included in-person delivery of feedback without motivational-interviewing and informing participants of their score on a psychological measure of gambling severity. In a population of college-students, Larimer et al. (2012) found that personalized feedback intervention (PFI) and cognitive–behavioral intervention (CBI) led to reductions in gambling among at-risk or probable pathological problem gamblers. They concluded that a single-session personalized feedback intervention and a multi-session cognitive–behavioral intervention may be helpful in reducing disordered gambling among US college students.

Neighbors et al. (2015) evaluated the impact of personalized normative feedback (PNF) in a sample of 252 U.S. college students. Personalized normative feedback (PNF) is a brief intervention designed to correct misperceptions regarding the prevalence of problematic behavior by showing individuals engaging in such behaviors that their own behavior is atypical with respect to actual norms. Their results supported the use of PNF as a standalone brief intervention for at-risk gambling students. They also concluded that the intervention effects were moderated by self-identification with other student gamblers, suggesting that PNF works better at reducing gambling for those who more strongly identified with other student gamblers.

Several studies have found promising results with regard to brief telephone and workbook interventions for individuals with gambling problems (e.g., Abbott et al., 2012, 2018; Hodgins et al., 2011). The phone-calls in these studies used a motivational interviewing approach which encouraged gamblers to think about their gambling. In a review of the existing literature, Yakovenko et al. (2015) concluded that motivational interviewing was associated with significant reduction in gambling frequency up to a year after treatment delivery. They also found that for gambling expenditure, motivational interviewing yielded significant reductions in the amount of money spent gambling compared to players who were not treated with motivational interviewing but this was at post-treatment only.

Only one previous study investigated the impact of personalized behavioral feedback by telephone and letters on actual gambling expenditure in a sample of real-world gamblers (i.e., Jonsson et al., 2019). In this study, a sample of 1,003 matched triplets was selected from the top 0.5% of gamblers based upon their annual expenditure. Gamblers were randomly assigned to the feedback intervention by telephone, letter, or a no-contact control condition. The study found that over 12 weeks, theoretical loss^1^ decreased 29% for the phone-call group and 15% for the letter group, compared to 3% of the control group. A positive effect of the follow-up contact was limited to participants who at the initial call indicated an interest in receiving a follow-up call. Jonsson et al. (2019) concluded that contacting high-spending players about their gambling expenditure appeared to be an effective method for gambling companies to meet their duty to care for their customers.

### Gambling in the Netherlands

In 2021, the Netherlands (where the present study was carried out) introduced a new gambling law which also includes licensed online gambling. Since October 2021, gambling operators have been legally allowed to offer casino games as well as sports betting online for Dutch residents. The regulation includes a number of player protection policies. Amongst others, license holders have to monitor gambler behavior for indications of problematic gambling. They are also obliged to interact with gamblers in case of a positive identification of problem gambling. In a review of problem gambling worldwide Calado and Griffiths (2016) cited two Dutch prevalence studies. The first one comprised 5,575 participants aged 16 years and over (Bruin et al., 2006). The findings showed that 1% were probable pathological gamblers (SOGS [South Oaks Gambling Screen] 5+) and 1.5% were potential problem gamblers (score of 3–4 in SOGS) – both lifetime prevalence rates. The past-year prevalence rates for pathological and problem gambling were 0.3% and 0.6%, respectively. The highest prevalence of problem gambling was present among males, among individuals aged between 30 and 50 years and between 18 and 30 years, among ethnic minorities, and among the unemployed.

The second prevalence survey was conducted in 2011 by Bieleman et al. (2011) comprising approximately 6,000 participants. The percentage of problem gambling (5 + on the SOGS) was 0.15% and the prevalence of at-risk gambling (3–4 SOGS) was 0.68%. Moreover, the prevalence of recreational gamblers (< 3 in SOGS) was 64.4%. The rates of at-risk and problem gambling did not change statistically between 2005 and 2011 (see also Goudriaan 2014). The authors are not aware of any Dutch prevalence studies in the past decade or since the introduction of online gambling in October 2021. Ipsos Research (2022) conducted a study after the introduction of online gambling in the Netherlands and found that the number of Dutch people who had played online in the past 12 months remained the same compared to the previous year (i.e., approximately 11% of the adult population). Of the Dutch who played online since 1 October 2021, 78% had only done so with providers licensed in the Netherlands.

### The Present Study

Personalized feedback interventions (PFIs) are becoming a common practice among online gambling operators (Harris & Griffiths, 2017). Furthermore, most European regulations require online gambling operators to interact with gamblers in case of high expenditure or indications of problematic gambling. However, there is limited research about the effects of PFIs. The present study aimed to provide insight on the impact of PFIs on subsequent gambling behavior among a Dutch sample of real-world gamblers.

## Method

### Participants

*Nederlandse Loterij* (the national Dutch Lottery operator which offers sports-betting and casino on the website *toto.nl*) provided access to a secondary dataset comprising tracking data from online casino and sports betting gamblers (N = 2,576) who were contacted either by e-mail or telephone between November 2021 and March 2022 if they showed signs of problematic gambling as identified using behavioral tracking software. Players who were contacted multiple times by e-mail, telephone or both were excluded from the analysis in order to be able to assign the effect to one type of contact. Of the 2,576 gamblers, 1,876 were contacted by e-mail and 700 were contacted by telephone. The average age of participants was 41.73 years (SD = 13.34) and 34% of the sample were females (N = 874). *Nederlandse Loterij* utilizes *Mentor*, a commonly used behavioral tracking tool used for the identification of problematic gambling (Auer & Griffiths, 2020). *Mentor’s* risk classification was part of the process which led to the selection the contacted gamblers. The present authors evaluated the extent to which the contacts by email or telephone had an effect on their subsequent gambling behavior.

### Rationale for Matched Pairs Design

The main aim of the present study was to determine whether the receiving of an e-mail or a telephone call by the gambling operator had an effect on subsequent playing behavior compared to those gamblers who were not contacted. However, it is not appropriate to simply compute the behavioral change after the contact. After the dataset was provided, the present authors gave very careful consideration to all of the ways in which the data could be analyzed. Following an initial inspection of the data, it became clear that analyzing the behavioral change before and after the gamblers who were contacted (i.e., within-group analysis) would not be particularly meaningful because there was very large variation in the amount of time and money that the individuals’ spent gambling. For instance, some gamblers spent a lot of money gambling every day before being contacted while others spent comparably little. The resulting mean average differences in terms of money spent gambling would likely be spurious because of the large individual differences in gambling behavior. Furthermore, there was no way of assessing whether the difference in the amount of money spent gambling within group was statistically significant because there was no reliable comparison point. Therefore, a control group was needed.

One way to determine a valid control group is via a matched pairs design in which similar gamblers out of the population are assigned to each of the 2,576 target group members who were contacted between November 2021 and March 2022. Similarity with respect to wagering and depositing was computed based on the 30 days prior the contact. In the remainder of the present paper, the 30 days prior to contact being made with the gambler is referred to as time period 1 (T1), and the 30 days after contact was made with the gambler is referred to time period 2 (T2). The control group population only comprised gamblers that were not contacted but who were most similar to the target group with respect to their behavior between November 2021 and March 2022. Matched pairs for the target group members were chosen using the following criteria and was very similar to the procedures employed in previous studies (e.g., Auer & Griffiths 2015a; Auer et al., 2018):

- *Age*: Control group members had to be at most five years younger or older as the target group member.
- *Gender*: Control group members had to be the same gender as the target group member for matching purposes.
- *Amount wagered 30 days prior to contact*: Control group members had to have the same amount wagered as the target group. For instance, if a target group member’s amount wagered was €1,000, the control group member’s amount wagered needed to be within €900 to €1,100 in order to be considered for matching purposes.
- *Amount deposited 30 days prior to the contact*: Control group members had to have the same amount deposited as the target group. For instance, if a target group member’s amount deposited was €1,000, the control group member’s amount deposited needed to be within €900 to €1,100 in order to be considered for matching purposes.

This matching procedure ensured that a target group member was assigned one or more control group members only if the monetary gambling intensity and demographic profile was most similar. All of the four criteria in the present study (i.e., age, gender, amount wagered, amount bet) were weighted equally. For that reason, each target group member was matched with none, or one or more control group members (as described above). Out of the 2576 target group gamblers who had received at least one feedback message during November 2021 and March 2022, 1,592 gamblers (62%) were assigned at least one control group member who was not contacted. Therefore, 38% of the target group members did not match any control group member with respect to the four criteria. This is similar to that reported by Auer et al. (2020) who also utilized a matched-pairs design and reported that 40% of target group gamblers could not be matched to any control group member. Unmatched gamblers were subsequently discarded from the analysis.

If a target group member was matched with several control group members, the average amount wagered and amount deposited in the 30 days before the contact date was computed for all the matched control group gamblers for this specific target group member. The matched gamblers were therefore aggregated to one “virtual” control group gambler for each target group gambler. In order to determine the effect for each gambler, the amount of money wagered, amount of money deposited, number of monetary deposits, amount of time spent gambling, and gambling frequency (i.e., number of gambling days) in T2 was divided by the same metrics in T1. This indicator was the ‘ratio’.

Consequently, the smaller the ratio, the lower the subsequent gambling intensity (in terms of the amount of money wagered, amount of money deposited, number of monetary deposits, amount of time spent gambling, and gambling frequency), and therefore the higher the effect of the contact made with the gambler. Each target group member’s computed ratio was compared to the ratio of the respective virtual matched pairs gambler for each of the five metrics relating to the time and money spent gambling. If a target group member’s ratio was smaller than the respective control group’s ratio it was concluded that the target group member’s behavior decreased more as a consequence of the contact compared to the control group members who were not contacted. Therefore, for each target/control pair, binary variables were computed with respect to each of the five metrics. The actual difference was not analyzed because the different target/control pairs showed large individual variation. The way the study was designed was to ensure that the gambling behavior between the two groups were comparable (and is why the matched pairs design was chosen).

### Statistical Analysis

The authors tested whether the five metrics relating to the time and money spent gambling followed a normal distribution according to D’Agostino (1971). Nonparametric Kruskal Wallis tests were used for group comparisons (Kruskal, 1952). The authors used the programming language *Python* (Van Rossum, 2007) to analyze the dataset. The amount of money deposited (K = 3033, *p* < 0.001), amount of money wagered (K = 2140, *p* < 0.001), number of monetary deposits (K = 2603, *p* < 0.001), time spent gambling (K = 383, *p* < 0.001), and number of gambling days (K = 2164, *p* < 0.001) in the 30 days prior to being contacted significantly deviated from a normal distribution. The effect of e-mail or phone contacts made by the gambling operator was analyzed with respect to the five metrics relating to time and money spent gambling. Gambling frequency was measured using the number of distinct days on which at least one wager was placed. A significance level of 1% was used for statistical testing.

## Results

### Matched-pairs Analysis

A total of 2,576 gamblers were either contacted by e-mail or called by the gambling operator between November 2021 and March 2022. Out of the 2,576 gamblers, 1,592 were matched with at least one gambler from the control group. Table 1 reports age, gender, amount of money deposited, and amount of money wagered 30 days prior to being contacted for gamblers who were matched with any control group member and those who were not. In the group of gamblers who received an e-mail the ones who were matched were younger and less frequently female. The corresponding Mann-Whitney U-Test regarding the age difference (U = 636,321, *p* < 0.001) and the z-test regarding the gender difference (z=-12.89, *p* < 0.001) were both significant.

Gamblers who were matched (compared to those who were not matched) deposited less money and wagered less money in the 30 days prior to the e-mail. The corresponding Kruskal-Wallis tests were significant (K = 634236.5, *p* < 0.001; K = 645,387, *p* < 0.001). The same pattern was observed for the group of gamblers who were not matched. The corresponding statistical tests for age (U = 58,381, *p* < 0.001), gender (Z=-5.34, *p* < 0.001), amount of money deposited (U = 42,313, *p* < 0.001), and wagered (U = 42,755, *p* < 0.001) were all significant.

Table 1 also shows higher monetary deposits and wagers for gamblers who were called by telephone compared to gamblers who received an e-mail. This was the case for both matched and unmatched gamblers. Furthermore, the mean values for the amount of money wagered and deposited were larger than the corresponding median values. Gamblers who were called by telephone and matched with at least one control group member deposited on average €15,938 in the 30 days prior to the call. The corresponding median value was €12,516. This large difference between mean and median means that a small number of gamblers generated a large amount of money deposited and wagered, respectively.

**Table 1:** Demographic distribution and spending 30 days prior to e-mail/telephone contact for gamblers who were matched and gamblers who were not be matched with any control group member

			Age (years)		Female		Amount deposited (€)		Amount wagered (€)	
Matched	Contact	N	Mean	SD	N	%	Mean	Median	Mean	Median
No	E-mail	668	42.56	12.61	355	53%	7497	5296	63,881	40,566
Yes	E-mail	1208	39.31	10.45	313	26%	5036	4081	37,832	29,988
No	Telephone	316	40.56	12.15	125	40%	15,938	12,516	151,159	91,609
Yes	Telephone	384	37.26	9.49	81	21%	8265	7150	60,736	48,345
		2576	40.11	11.27	874	34%	5912	4431	47,107	32,543

On average, each target group member was matched with eight control group gamblers. The minimum number of matches was one and the maximum was 158. The size of the control group was 369,961 gamblers. The assignment of multiple controls to one target group member was based on the recommendations of Miettinen (1969). More recently, Ming and Rosenbaum (2000) noted that matching with a fixed number of controls may remove only 50% of the bias in a covariate, whereas matching a variable with many controls may remove up to 90% of the bias.

### Global Effect of e-mail vs. Telephone Contact by the Gambling Operator

The effect that personalized contacts had on subsequent monetary depositing, monetary wagering, deposit frequency, gambling time duration, and gambling frequency of those who were contacted by the gambling operator was statistically analyzed and compared with that of the control group. It was assumed that any difference between the gambling behavior in the two groups could be due to chance and would be similar to the tossing of a coin. For that reason, it was assumed under the null hypothesis that in 50% of the cases the target group’s gambling behavior (as measured by the amount of time and money spent) would be higher than the control group’s gambling behavior and in 50% of the cases the control group’s gambling behavior (as measured by the amount of time and money spent) would be higher than the target group’s gambling behavior. Therefore, it was assumed that any deviation from the distribution would be due to the effect of being contacted by the gambling operator. Consequently, the difference between the actual observed percentage to the expected percentage (50%) of gambling behavior was statistically examined.

Of the 1,208 matched target group members who were contacted via e-mail (and compared to the ratio of the matched control group members), 750 showed a smaller amount of money deposited ratio (62%), 727 showed a smaller amount of money wagered ratio (60%), 671 showed a smaller number of monetary deposits ratio (56%), 655 showed a smaller time spent gambling ratio (54%), and 596 showed a smaller gambling frequency ratio (49%). Except for gambling frequency, gambling behavior decreased more among the group of gamblers who were contacted by email by the gambling operator compared to the matched control group members. The resulting ratios reported above were compared to the expected ratio of 50% using a z-test. The results showed significant differences for amount of money deposited (z = 8.66; *p* < 0.001), amount of money wagered (z = 7.23, *p* < 0.001), number of monetary deposits (z = 3.88, *p* < 0.001) and time spent gambling (z = 2.95, *p* = 0.0032). The 49% ratio reported for playing frequency did not significantly deviate from the expected ratio of 50% (z=-0.46, *p* = 0.65). Therefore, the e-mail contact had the desired impact on subsequent playing behavior with respect to monetary spend and time spend, but not gambling frequency.

Of the 384 matched target group members who were contacted by telephone (and compared to the ratio of the matched control group members), 252 showed a smaller amount of money deposited ratio (66%), 240 showed a smaller amount of money wagered ratio (62%), 241 showed a smaller number of monetary deposits ratio (63%), 226 showed a smaller gambling duration ratio (59%), and 213 showed a smaller gambling frequency ratio (55%). Gambling behavior decreased more among the group of gamblers who were contacted by telephone by the operator compared to the matched control group members with respect to all five behavioral metrics. The resulting ratios reported above were compared to the expected ratio of 50% using a z-test. The results showed significant differences for amount of money deposited (z = 6.45; *p* < 0.001), amount of money wagered (z = 5.06, *p* < 0.001), number of monetary deposits (z = 5.17, *p* < 0.001), gambling time duration (z = 3.53, *p* = 0.0004) and gambling frequency (z = 2.15, *p* = 0.03). The 55% ratio reported for gambling frequency did not significantly deviate from the expected ratio of 50% (z=-0.46, *p* = 0.65). Therefore, the telephone contact had the desired impact on subsequent playing behavior with respect to monetary spend and time spend, but not gambling frequency.

### Effect by Gambling Operator Contact type

Table 1 shows that gamblers who were contacted by telephone wagered and deposited more money than gamblers who were contacted by e-mail. Table 2 shows the effects of the five metrics with respect to the control group by contact type. The average effects were consistently larger in the group of gamblers who were contacted by telephone. However, none of the differences were statistically significant.

**Table 2:** Effects by contact type (e-mail vs. telephone)

	E-mail	Telephone	
N	1208	384	1592
Amount deposited ratio	62%	66%	z=-1.25, *p* = 0.21
Amount wagered ratio	60%	62%	z=-0.81, *p* = 0.41
Number of deposits ratio	56%	63%	z=-2.49, *p* = 0.013
Gambling duration ratio	54%	59%	z=-1.59, *p* = 0.11
Gambling frequency ratio	49%	55%	z=-2.10, *p* = 0.036

### Effect of Gambling Operator Contact by Gambling Intensity

Analysis was also carried out to see if gambling intensity was associated with the effect of the contact by the gambling operator. To do this, the target group members were divided into four equally sized groups according to their amount of money wagered in the 30 days before the contact. This was done separately for the gamblers who received an e-mail and gamblers who received a telephone call. Table 3 shows the effects in the e-mail group with respect to the five metrics in each of four intensity groups. Chi-square tests showed that none of the five metrics’ effects were significantly different between the four intensity groups. Kruskal-Wallis tests also showed that the four intensity groups did not differ with respect to age. However, there was a significant difference with respect to gender. The percentage of females (14%) was lowest among the 25% of gamblers who wagered the most money in the 30 days prior to the e-mail contact. The percentage of females did not vary meaningfully in the other three groups. The overall average percentage of females among gamblers who received e-mail contact from the gambling operator and were matched was 26%.

**Table 3:** Effects of e-mail contact in four intensity groups based on amount wagered 30 days prior to contact by the gambling operator

Quantile amount wagered	1	2	3	4	
N	302	302	302	302	1208
Amount deposited ratio	64%	62%	62%	61%	χ^2^ = 0.59, *p* = 0.90
Amount wagered ratio	59%	59%	62%	61%	χ^2^ = 0.98, *p* = 0.81
Number of deposits ratio	54%	58%	57%	53%	χ^2^ = 0.59, *p* = 0.90
Gambling duration ratio	51%	55%	56%	54%	χ^2^ = 1.61, *p* = 0.66
Gambling frequency ratio	44%	51%	50%	53%	χ^2^ = 5.48, *p* = 0.14
Age	39.88 (SD = 11.02)	39.81 (SD = 10.91)	39.52 (SD = 10.50)	38.03 (SD = 9.18)	K = 3.64, *p* = 0.30
Female	94 (31%)	91 (30%)	84 (29%)	41 (14%)	χ^2^ = 32.34, *p* < 0.001

Table 4 shows the effects of receiving a telephone call from the gambling operator with respect to the five metrics in each of the four intensity groups. The four groups were based on the amount of money wagered 30 days prior to receiving a telephone call. Chi-square tests showed that none of the five metrics’ effects were significantly different between the four intensity groups. Kruskal-Wallis tests also showed that the four intensity groups were not significantly different with respect to age. However, there was a significant difference with respect to gender. The percentage of females (7%) was lowest in the 25% of gamblers who wagered the most money in the 30 days prior to receiving an e-mail. The percentage of females was largest (34%) in the 25% of gamblers who wagered the least amount of money. The overall average percentage of females among gamblers who received e-mail contact from the gambling operator and were matched was 21%.

**Table 4:** Effects of telephone contact by the gambling operator in four intensity groups based on amount of money wagered 30 days prior to contact

Quantile amount wagered	1	2	3	4	
N	96	96	96	96	384
Amount deposited ratio	66%	67%	71%	59%	χ^2^ = 2.86, *p* = 0.41
Amount wagered ratio	62%	60%	62%	65%	χ^2^ = 0.35, *p* = 0.95
Number of deposits ratio	67%	64%	67%	54%	χ^2^ = 1.90, *p* = 0.60
Gambling duration ratio	60%	56%	59%	59%	χ^2^ = 0.39, *p* = 0.94
Gambling frequency ratio	56%	52%	55%	58%	χ^2^ = 0.79, *p* = 0.85
Age	37.49 (SD = 11.97)	34.14 (SD = 8.74)	36.59 (SD = 8.00)	37.80 (SD = 8.88)	K = 0.9, *p* = 0.82
Female	33(34%)	21 (22%)	20 (21%)	7 (7%)	χ^2^ = 21.20, *p* < 0.001

### Effect of Gambling Operator Contact by Gender

Analysis was also carried out to see if gender was associated with the effect of the contact by the gambling operator. Table 5 shows the effects of the contact by e-mail for females and males. Except for gambling frequency, the effects were larger among males. However, none of the respective z-tests were significant.

**Table 5:** Effects of gambling operator e-mail contact by gender

	Female	Male	
N	313	895	1208
Amount deposited ratio	57%	64%	z=-2.35, *p* = 0.019
Amount wagered ratio	54%	62%	z=-2.47, *p* = 0.014
Number of deposits ratio	54%	56%	z=-0.77, *p* = 0.43
Playing duration ratio	52%	55%	z=-0.88, *p* = 0.38
Playing frequency ratio	53%	48%	z = 1.39, *p* = 0.17

Table 6 shows the effects of receiving a telephone call from the gambling operator for females and males. The effects were larger among males compared to females. However, none of the respective z-tests were significant.

**Table 6:** Effects of gambling operator telephone contact by gender

	Female	Male	
N	81	303	384
Amount deposited ratio	58%	68%	z=-1.62, *p* = 0.10
Amount wagered ratio	60%	63%	z=-0.42, *p* = 0.68
Number of deposits ratio	59%	64%	z=-0.73, *p* = 0.17
Gambling duration ratio	52%	61%	z=-1.44, *p* = 0.15
Gambling frequency ratio	54%	56%	z=-0.23, *p* = 0.82

### Effect of Gambling Operator Contact by age

In order to determine whether the effect of gambling operator contact varied across age, the authors classified gamblers into five age groups as shown in Tables 7 and 8. Table 7 reports the effects of receiving e-mail contact by the gambling operator for each age group. None of the five metrics was significantly different between the age groups. There was also no clear pattern with respect to the size of the effects in the different age groups. Effects were neither descending or ascending in the same way across age groups.

**Table 7:** Effects of gambling operator e-mail contact by age group

Age group (in years)	<=33	34–43	44–53	54–63	>=64	
N	428	399	242	115	24	1208
Amount deposited ratio	64%	64%	57%	61%	58%	χ^2^ = 4.56, *p* = 0.33
Amount wagered ratio	64%	62%	56%	52%	54%	χ^2^ = 8.07, *p* = 0.09
Number of deposits ratio	57%	56%	51%	61%	50%	χ^2^ = 3.7, *p* = 0.45
Gambling duration ratio	56%	55%	52%	50%	46%	χ^2^ = 2.52, *p* = 0647
Gambling frequency ratio	49%	50%	47%	54%	42%	χ^2^ = 2.39, *p* = 0.66

Table 8 shows the respective numbers for gamblers which were contacted by telephone by the gambling operator. None of the effects were significant between the age groups. There was also no clear pattern with respect to the distribution across age groups. Only one gambler was at least 64 years old among the gamblers who were contacted by telephone.

**Table 8:** Effects of gambling operator telephone contact by age group

Age group (in years)	<=33	34–43	44–53	54–63	>=64	
N	156	133	68	26	1	384
Amount deposited ratio	63%	65%	72%	65%	100%	χ^2^ = 2.15, *p* = 0.71
Amount wagered ratio	62%	59%	72%	54%	100%	χ^2^ = 4.63, *p* = 0.33
Number of deposits ratio	65%	58%	71%	54%	100%	χ^2^ = 4.86, *p* = 0.30
Gambling duration ratio	60%	57%	65%	46%	100%	χ^2^ = 3.58, *p* = 0.47
Gambling frequency ratio	57%	52%	60%	50%	100%	χ^2^ = 2.60, *p* = 0.63

## Discussion

The present study investigated the effects of online casino and sports-betting gamblers being contacted by *Nederlandse Loterij* either via e-mail or telephone call. The reason for the contact was showing signs of problematic gambling which were identified via the player tracking tool *Mentor*. Gamblers were not randomly assigned to one of the two groups. Consequently, the authors chose a matched-pairs design to create a comparable control group. Matched-pairs designs are commonly used to study causal effects in retrospective studies and in situations where a randomized experimental set-up is not possible (e.g., Cummings et al., 2002; Freedman et al., 1997). During the process, the 2576 target group gamblers were matched with similar gamblers based on the amount of money wagered, amount of money deposited, age, and gender. The control group gamblers were not contacted. Of the 2,576 gamblers who were contacted by the gambling operator, only 1,592 could be matched with one control group (62%). This percentage is similar to the one reported by Auer et al. (2020) in their study of a loss-limit reminder among Norwegian online gamblers. They also applied a matched-pairs design because of the lack of randomized assignment. Similar to Auer et al. (2020), gamblers who were not matched wagered more money and deposited more money in the 30 days prior to being contacted by the gambling operator. This was the case for both gamblers who received an e-mail or received a telephone call. *Nederlandse Loterij*’*s* main reason for the contact were signs of problematic gambling and the authors assumed that the majority of the most intense gamblers were therefore contacted. This explains why the non-matched gamblers displayed higher gambling intensities.

Among the 1,208 matched gamblers who received an e-mail from the gambling operator, a significant reduction in amount of money deposited, amount of money wagered, number of monetary deposits, and time spent gambling were observed in the 30 days after being contacted. Gambling frequency as measured by the number of gambling days did not change significantly. The same results were observed among the 384 matched gamblers who received a telephone call from the gambling operator. The findings support the only previous comparable study by Jakobsson et al. (2019). They applied a fully randomized experimental design and also found that high-intensity gamblers who received a letter or a telephone call from the gambling operator (i.e., *Norsk Tipping*) subsequently reduced their monetary gambling expenditure. Although the group that received a telephone call displayed larger reductions in their gambling than those who received e-mails, the differences were not statistically significant. This contradicts the findings by Jakobsson et al. (2019) who found that telephone calls were more efficient than letters (although letters are not the same as emails even though they are both print interventions). The present authors hypothesize that the lack of significance between the e-mail and the telephone call group could also be due to the small sample size.

The present study also tested whether the effects of being contacted by the gambling operator varied across gambling intensity. Gamblers contacted by e-mail as well as those contacted by telephone were divided into four groups based on the amount of money wagered in the 30 days prior to being contacted. None of the effects were significantly different between the four intensity groups. In their real-world matched-pairs study, the participants in Auer and Griffiths’ (2015a) study were provided with personalized feedback. However, there was no correlation between gambling intensity and the effect of personalized feedback in that study.

There was a significant correlation between gender and gambling intensity. Females were less likely to be among the most intense gamblers. This holds true for gamblers in the group that received an e-mail from the gambling operator as well as in the group that received a telephone call. A number of previous studies have found that males gamble more intensely or having a higher likelihood of problem gambling than females (e.g., Ivanova et al., 2019; Husky et al., 2015; Kairouz et al., 2016). With the exception of gambling frequency, the effects were larger among males than among females. However, none of the differences were significantly different. A larger sample size would most likely have led to significant results. The authors also wanted to test if there were any age differences with respect to the effects of the contact. However, no significant differences or obvious patterns were observed.

### Limitations

The present study is only the second attempt to investigate the effect of an online gambling operator contacting gamblers displaying potentially problematic gamblers. Despite the many strengths of this study, there are a number of limitations. *Nederlandse Loterij* selected the high-risk gamblers who were contacted either by telephone or by e-mail. For that reason, not all high-risk gamblers could be matched with a control group member because the highest spending gamblers were all selected to be contacted. This creates a bias and the conclusions do not apply to the highest spenders. This is simply a consequence of the thoroughness of the operators safer gambling procedures and the regulatory requirements. One of the major limitations of the present study was that data were only collected from one gambling environment in one particular country (Netherlands). Replicating the results with other online gambling operators’ websites from different countries would help further corroborate the findings reported here. Another limitation is that there is no way of knowing whether the target group gambled with other online operators during the experimental period. Studies such as the British Gambling Prevalence Surveys (Wardle et al., 2007, 2011) have shown that at-risk and problem gamblers in particular engage with numerous gambling websites and gambling forms. Not being able to confirm such assertions through self-report methodologies is arguably another limitation of the study. There is also the possibility that more than one person gambled using the same account (e.g., a husband and wife) although the number of instances where this occurred is likely to be low. Future studies could examine the impact on gambling behavior by comparing the impact of one message reminder compared to multiple reminders.

## Conclusion

The present study found that online gamblers reduced both the money and time spent gambling when contacted either by an e-mail or telephone call by the online gambling operator. This is an important finding, given that regulators increasingly require operators to interact with gamblers and evaluate the effects of such personalized interactions. The study also showed that e-mails appear to be as effective as telephone calls. E-mails require less personnel than telephone calls which means that more gamblers can be informed and the information could also be tailored to a gambler’s individual gambling patterns. The results also showed that a reduction of gambling expenditure on both time and money was as likely among high-spending gamblers as among low-spending gamblers.

The present study is the latest in a growing number of studies that have evaluated the efficacy of responsible gambling tools in real world settings using real gamblers engaging in real time gambling on real gambling websites (as opposed to efficacy evaluations in laboratory situations where the sample sizes are often very small and not necessarily representative of real gamblers because of the use of convenience sampling). The results of the present study are of use to many different stakeholder groups including researchers in the gambling studies field (who can attempt to replicate and extend the present study in other jurisdictions and cultures), and the gambling industry (who can employ such responsible gambling tools knowing there is an empirical base demonstrating their efficacy), as well as regulators and policymakers who can recommend or enforce that gambling operators utilize responsible gambling tools such as contacting those who may be displaying problematic gambling behaviors as a way of minimizing harm and protecting gamblers.

## Electronic Supplementary Material

Below is the link to the electronic supplementary material.

Supplementary Material 1

## Data Availability

The data for this study are not available due to commercial sensitivity.

## Footnotes

1 Theoretical loss is computed as amount of money wagered multiplied by the house advantage for each game which was played (Auer et al., 2012).
