# Supplementary material for: The Impact of Personalized Feedback Interventions by a Gambling Operator on Subsequent Gambling Expenditure in a Sample of Dutch Online Gamblers
Source: J Gambl Stud. 2022 Nov 9;39(2):929–46. doi: 10.1007/s10899-022-10162-2 (PMC10175399; doi:10.1007/s10899-022-10162-2)
Supplement: Supplementary file 1 — Supplementary Material 1 [file 10899_2022_10162_MOESM1_ESM.doc]

#### Dear Dr. Grant,

Please find attached a novel paper entitled *‘The impact of personalized feedback interventions by a gambling operator on subsequent gambling expenditure in a sample of Dutch online gamblers’* which Dr. Michael Auer and I are submitting for publication in the *Journal of Gambling Studies*.

Our new study provides insight on the impact of personalized feedback interventions (PFIs) on subsequent gambling behavior among a Dutch sample of real-world gamblers. *Nederlandse Loterij* (the national Dutch Lottery operator) gave us access to a secondary dataset comprising tracking data from online casino and sports betting gamblers (N=2,576) who were contacted either by e-mail or telephone between November 2021 and March 2022 if they showed signs of problematic gambling as identified using behavioral tracking software. Compared to matched controls (n=369,961 gamblers), we found that Dutch gamblers who received a PFI (via e-mail [n=1876] or a telephone call [n=700]) from the gambling operator had a significant reduction in amount of money deposited, amount of money wagered, number of monetary deposits, and time spent gambling in the 30 days after being contacted. The results of our study are of use to many different stakeholder groups including researchers in the gambling studies field and the gambling industry as well as regulators and policymakers who can recommend or enforce that gambling operators utilize responsible gambling tools such as using PFIs to those who may be displaying problematic gambling behaviors as a way of minimizing harm and protecting gamblers.

- The work described is this paper has not been published previously, that it is not under consideration for publication elsewhere.
- Both authors have mutually consented to submit the manuscript to Journal of Gambling Studies
- The manuscript is original work of both authors.

Thank you and kind regards.

Yours sincerely,

Dr. Mark Griffiths

Distinguished Professor of Behavioural Addiction

Director, International Gaming Research Unit, Psychology Department

Nottingham Trent University

50 Shakespeare Street, Nottingham, NG1 4FQ

[mark.griffiths@ntu.ac.uk](mailto:mark.griffiths@ntu.ac.uk)
